# Paradoxical Sleep Deprivation Causes Cardiac Dysfunction and the Impairment Is Attenuated by Resistance Training

**DOI:** 10.1371/journal.pone.0167029

**Published:** 2016-11-23

**Authors:** Sara Quaglia de Campos Giampá, Marcos Mônico-Neto, Marco Tulio de Mello, Helton de Sá Souza, Sergio Tufik, Kil Sun Lee, Marcia Kiyomi Koike, Alexandra Alberta dos Santos, Ednei Luiz Antonio, Andrey Jorge Serra, Paulo José Ferreira Tucci, Hanna Karen Moreira Antunes

**Affiliations:** 1 Programa de Pós-Graduação Interdisciplinar em Ciências da Saúde, Universidade Federal de São Paulo, Campus Baixada Santista, Santos-SP, Brazil; 2 Laboratório Interdisciplinar em Fisiologia e Exercício, LAIFE, São Paulo-SP, Brazil; 3 Departamento de Psicobiologia, Universidade Federal de São Paulo, Campus São Paulo, São Paulo-SP, Brazil; 4 Departamento de Bioquímica, Universidade Federal de São Paulo, Campus São Paulo, São Paulo-SP, Brazil; 5 Departamento de Clínica Médica, Laboratório de Emergências Clínicas, Universidade de São Paulo, São Paulo – SP, Brazil; 6 Programa de Pós-Graduação em Ciências da Saúde, Instituto de Assistência Médica do Servidor Público Estadual, São Paulo – SP, Brazil; 7 Departamento de Medicina, Disciplina de Cardiologia, Universidade Federal de São Paulo, Campus São Paulo, São Paulo-SP, Brazil; 8 Programa de Pós-Graduação em Ciências da Reabilitação e Programa de Pós-Graduação em Biofotônica Aplicada às Ciências da Saúde, Universidade Nove de Julho, São Paulo-SP, Brazil; 9 Departamento de Biociências, Universidade Federal de São Paulo, Campus Baixada Santista, Santos-SP, Brazil; Maastricht University, NETHERLANDS

## Abstract

**Background:**

Paradoxical sleep deprivation activates the sympathetic nervous system and the hypothalamus-pituitary-adrenal axis, subsequently interfering with the cardiovascular system. The beneficial effects of resistance training are related to hemodynamic, metabolic and hormonal homeostasis. We hypothesized that resistance training can prevent the cardiac remodeling and dysfunction caused by paradoxical sleep deprivation.

**Methods:**

Male Wistar rats were distributed into four groups: control (C), resistance training (RT), paradoxical sleep deprivation for 96 hours (PSD96) and both resistance training and sleep deprivation (RT/PSD96). Doppler echocardiograms, hemodynamics measurements, cardiac histomorphometry, hormonal profile and molecular analysis were evaluated.

**Results:**

Compared to the C group, PSD96 group had a higher left ventricular systolic pressure, heart rate and left atrium index. In contrast, the left ventricle systolic area and the left ventricle cavity diameter were reduced in the PSD96 group. Hypertrophy and fibrosis were also observed. Along with these alterations, reduced levels of serum testosterone and insulin-like growth factor-1 (IGF-1), as well as increased corticosterone and angiotensin II, were observed in the PSD96 group. Prophylactic resistance training attenuated most of these changes, except angiotensin II, fibrosis, heart rate and concentric remodeling of left ventricle, confirmed by the increased of NFATc3 and GATA-4, proteins involved in the pathologic cardiac hypertrophy pathway.

**Conclusions:**

Resistance training effectively attenuates cardiac dysfunction and hormonal imbalance induced by paradoxical sleep deprivation.

## Introduction

In plurality, sleep is defined as a vital state of homeostatic regulation with specific behavioral and electrophysiological characteristics. Whose sensory feedback and motor capacity is reduced, differing from coma or anesthesia by its rapid reversibility [1,2]. Sleep is divided into two phases, non- rapid eye movements (NREM) and rapid eye movements (REM). The former is associated with progressive reduced neuronal activity with three defined phases: N1, N2, N3 (slow wave sleep) [3]. The latter, is characterized by vivid dreaming and a high level of brain activity despite the muscular atony that suggests a deep sleep. This phase is also known as paradoxical sleep in rats [4,5].

Among the important functions of sleep, its role in the cardiovascular system has been highlighted in recent years. Part of this interest is related to the intriguing hemodynamic changes found during the period of sleep in contrast to those observed during wakefulness [6,7]. During NREM sleep, a reduction of cardiovascular activity is observed. On the other hand, during REM sleep this activity appears to be very similar to the cardiovascular acitivity observed during wakefulness [7,8]. Considering that the period of sleep is mainly composed of NREM, it is quite likely that this stage is related to a period of quiescence of the cardiovascular system. This is observed in the physiological variables such as heart rate (HR) and blood pressure (BP) [6].

Therefore, during sleep is observed an increased parasympathetic activity [8], whereas, sleep deprivation (SD) causes an increase in sympathetic activity and a decrease in parasympathetic activity [9]. These phenomena can be explained by changes in baroreflex sensitivity, which would result in an increase of BP and consequently cardiovascular changes [10–12]. Although, there is not a consensus yet for this plausible mechanism [11,13].

Regarding the endocrine axis, SD is also recognized for changing the secretion of hormones, increasing catabolic hormones (catecholamines and corticosterone) and reducing anabolic hormones (testosterone and Insulin-like growth factor-1(IGF-1)) [14,15]. These changes can influence cardiac cellular mechanisms such as Ca^2+^ handling proteins, which are involved in the maintenance of normal cardiac Ca^2+^ homeostasis and in the contractile function. Among these proteins, sarcoplasmic reticulum Ca^2+^ ATPase (SERCA2a) and its regulator phosphorylatable protein (phospho-Ser^16^-Thr^17^-phospholamban (PLN)), which in its dephosphorylated form (PLN) inhibits SERCA2a activity, both former and latter are responsible for Ca^2+^ uptake by the sarcoplasmic reticulum; ryanodine receptor (RyR), that is responsible for releasing the Ca^2+^ by the sarcoplasmic reticulum and; Na^+^/Ca^2+^ exchanger (NCX) involved in the Ca^2+^ extrusion by sarcolemma. [16–18].

When both, the hormones and the cardiac cellular mechanisms are altered they can increase the myocardium demands triggering the pathological cardiac hypertrophy, a compensatory adaptation to an increase in heart workload [19]. Pathological cardiac hypertrophy is associated with loss of myocites and fibrotic replacement, which impairs the cardiac function [20]. Angiotensin II (Ang II) and proteins such as nuclear factor of activated T-cells 3 (NFATc3) and GATA binding protein 4 (GATA-4) are factors associated with this remodeling process of the heart [20–22].

If SD can cause an autonomic, endocrine, moleculars and morphological disruption, thereby increasing the risk for cardiovascular diseases, there are strategies that may benefit the cardiac function. Among them, we highlight resistance training. Studies demonstrate that resistance training reduces resting BP and HR and triggers physiological concentric hypertrophy of cardiac tissue [23,24]. In addition, increased levels of IGF-1 and testosterone are also evident after resistance training [25,26]. Thus, the purpose of this study was to evaluate the effects of paradoxical sleep deprivation (PSD) on cardiac function in rats and to explore the benefits of resistance training.

## Materials and Methods

### Experimental Animals and Environmental Conditions

Wistar male rats aged 3 months and weighing 300–350 g at the beginning of the experiment were housed in groups of five inside standard polypropylene cages in a temperature-controlled (23±1°C) room with a 12:12 h light-dark cycle (light starting at 07:00 am) and were allowed free access to food and water. All procedures used in the present study complied with the Guide for the Care and Use of Laboratory Animals and the experimental protocol was approved by the UNIFESP Ethical Committee (#0764/10).

### Experimental Groups

The animals were distributed into four groups: 1) the control group (C) was maintained in their cages and was minimally manipulated during the experimental period (n = 30); 2) the resistance training group (RT) was composed of animals subjected to 8 weeks of resistance training (n = 30); 3) the PSD96 group consisted of animals submitted to PSD for 96 continuous hours (n = 30); and 4) the RT/PSD96 group was composed of animals subjected to 8 weeks of resistance training followed by the PSD for 96 continuous hours (n = 30).

### Resistance Training Protocol

Resistance training was performed with a ladder which is 110 cm high and 18 cm wide, with 2 cm intervals between the steps, and the stairs are placed at an 80° angle [27]. These specific dimensions and angulations facilitates the animals to climb to the top, where it is located a shelter (20x20x20 cm) for the animals to rest between climbing attempts.

The animals were allowed to familiarize themselves with the ladder for three consecutive days and then the maximum load was tested [27]. The animals had to climb the stairs carrying a load that was fixed to the base of the tail by self-fusing tape (Scotch^®^ 23 Rubber Tape-Scotch^®^ 3M) and was also connected to wires to increase the cylinders’ load as training progressed. During the training sessions, the animals were placed at the bottom of the ladder to climb the stairs 4–8 times while carrying a load, with 60-second intervals between the series. Animals took 8 to 12 steps to climb from the base to the top of the ladder [28].

For determining the load in the first week and start the resistance training protocol, we used the body weight as reference, thus animals were loaded with 50% of their body weight, and this load was gradually increased to 75%, 90% and 100% of the body weight on climbing attempts 2, 3 and 4, respectively. In subsequent climbing attempts, the load was increased by 30 g at each attempt until failure. Thus, to determine the new load in subsequent weeks of resistance training, we used the maximal carrying capacity determined in the previous maximal load test. The protocol was the same used with body weight, however, using the maximum load as reference. The sessions were conducted five times per week (Monday to Friday), and the Monday training session was replaced by the maximum load test to readjust the load. To avoid overtraining due to the high loads, prophylactic rest was introduced on Wednesday of weeks 6, 7, and 8 [28].

In order to evaluate the resistance training protocol efficiency on strength gains, and the importance of continuous physical exercise to acquire its benefits, we also evaluate the animals of C group. This group was submitted only to the session of maximum load determination, that occurred only once a week. With the data of the maximum load in C group we established a comparison with animals that trained 5 times per week, during 8 weeks.

### Paradoxical Sleep Deprivation

PSD was performed for 96 hours using the modified multiple platform method, which consisted of placing the rats inside a tiled water tank (123x44x44 cm), containing 14 circular platforms, 6.5 cm in diameter, with water within 1 cm of their upper surface. The rats could thus move around inside the tank by jumping from one platform to another. During paradoxical sleep, rats tend to fall off the platform due to muscular atonia and to wake upon contact with the water [29]. This SD method results in a complete loss of paradoxical sleep and promotes a 37% decrease in slow-wave sleep (a specific phase of NREM sleep) [30]. The water in the tank was changed daily. Prior to the protocol, the animals were placed on the platform for 1 h per day for 3 days for habituation.

### Euthanasia

Immediately after PSD or 48 h after the last training session, the animals were transferred to an adjacent room in a random order and were decapitated (between 08:00 and 10:00 am).

### Blood Sampling

For the blood sampling, 10 animals were used from each groups. Immediately after euthanasia, blood samples were collected and centrifuged to separate the plasma and serum and were stored at -80°C until the assays were performed. Serum testosterone levels were measured by a chemiluminescent enzyme immunoassay (*Unicell DXI 800*^®^, *Beckman Coulter*^®^, Brea, CA, USA). The plasma corticosterone concentrations were assayed using a commercial kit employing a double antibody radioimmunoassay specific for rats (MP Biomedicals^®^, Santa Ana, CA, USA). Plasma IGF-1 was assayed using a commercial ELISA kit specific for rats (USCN Life Science^®^, Houston, TX, USA). Serum T_3_ (triiodothyronine) and T_4_ (thyroxine) were measured by a chemiluminescent enzyme immunoassay (*Unicell DXI 800*^®^, *Beckman Coulter*^®^, Brea, CA, USA). C-reactive protein (CRP) concentrations were measured using a kinetic nephelometry method (IMMAGE^®^, Beckman Coulter^®^, Brea, CA, USA). Angiotensin II (Ang II) measurements was performed by the method of enzyme-linked immunosorbent assay using the specific commercial kit (Cloud-Clone Corp^®^, Houston, TX, USA).

### Histological Studies

#### *Plantaris* Muscle

*Plantaris* is the primary muscle used to climb the resistance training apparatus. The *Plantaris* muscle from the right leg of each rat was excised and dried for a few seconds using filter paper. The distal fragment of the *Plantaris* muscle was wrapped with a mixture of powdered milk and optimal cutting temperature compound (Tissue-Tek^®^ O.C.T. ^™^ Compound–Sakura, AJ Alphen aan den Rijn, The Netherlands, Europe) and serial cross-sections of 10 μm were obtained using a cryostat (Leica Microsystem-CM1850^®^, Nussloch, Germany, Europe) at -22°C. Then, the sections were placed on silanized glass slides. For the morphological analysis, the samples were subjected to hematoxylin-eosin (HE) staining and analyzed using a light microscope (Olympus BX50^®^, brightfield, and camera DP71- Melville, NY, USA) with 40x objective. The muscle fiber cross sectional area (CSA) was analyzed in a blind manner, and 300 fibers per muscle were counted using AxioVision 4.6 software (Carl Zeiss MicroImaging GmbH^®^, Jena, Germany, Europe). This parameter was assessed in six animals randomly chosen from each group.

### Heart Morphometric Study

After euthanasia, the hearts were arrested in diastole with 100 mM KCl and were washed with a solution of NaCl 0.9%. Both the atria and ventricles were dissected. The left atrium index (mg/mm) was calculated by normalizing then left atrium weight to the tibia length.

The left ventricle (LV) was cut in the equatorial plane. The middle to apex portion was fixed in 4% formaldehyde and embedded in paraffin, to cut into 4 μm sections. Tissue sections were stained with hematoxylin-eosin (HE) and Picro-Sirius red and were examined using a computerized image system (Leica QWin^®^ (version 3) software and Leica DFC^®^ 295 microscope, Cambridge, UK, Europe).

To estimate cardiomyocyte hypertrophy, HE-stained sections were examined under 40x objective to select transversally cut myocytes with central and visible nuclei. The CSA (μm^2^) of 20 cardiomyocites were calculated.

The collagen volume fraction was estimated in Picro-Sirius red stained sections under 40x objective. The collagen volume fraction was determined as the percentage of red-stained connective tissue areas per total myocardial area, excluding areas with fibrosis of the perivascular, endocardial and epicardial areas. For each animal, 20 visual fields of the myocardium were analyzed.

To determine the LV cavity diameter, digital images of whole LV were acquired using a light microscope (Olympus BX50^®^, brightfield, and camera DP71- Melville, NY, USA) with 1.25x objective. The LV cavity diameter was assessed with the AxioVision 4.6 software (Carl Zeiss MicroImaging GmbH^®^, Jena, Germany, Europe).

These analyses were assessed in six animals randomly chosen from each group.

### Western Blotting

Left ventricle (six animals per group, randomly selected) were homogenized using PBS (pH 7.2), containing Sigma-Aldrich, St. Louis, MO Complete Protease Inhibitor Cocktail Tablets (Roche Applied Science Inc. Penzberg–Germany) and PhosSTOP Phosphatase Inhibitor Cocktail Tablets (Roche Applied Science Inc. Penzberg–Germany). After four freezing and thawing cycles, the homogenate was centrifuged at 7,250 g for 5 minutes at 4°C; then, the supernatant was collected, and the pellet was discarded. The protein content of the supernatant was measured using BCA Protein Assay Reagent (Thermo Scientific Pierce Protein Biology). Aliquots from each sample were subjected to SDS-PAGE (4%, 6%, 10% or 20% depending on protein molecular weight) and transferred to a polyvinylidene fluoride (PVDF) membrane, which was incubated in a blocking solution (5% bovine serum albumin (BSA) in TBS-T (50 mM Tris, pH 7.4, 150 mM NaCl, and 0.1% Tween 20)) for 60–120 minutes at room temperature. The membrane was then incubated with primary antibodies diluted in blocking solution for two hours at room temperature and then rinsed three times (five minutes each) with TBS-T. Peroxidase-conjugated secondary antibody and luminol substrate (SuperSignal West Pico–Thermo Scientific Pierce Substrates) were used to detect the protein of interest. A digital image of the membrane was acquired using a gel documentation system (Uvitec: Cambridge—Alliance mini 4 m), and the band intensity was measured using commercial software (Uvitec^®^: Cambridge—UVIband).

The antibodies were used at the following dilutions: RyR (Thermo Scientific, 1:5000); SERCA2a (Cell Signaling, 1:1000); PLN (Cell Signaling, 1:1000); phospho-Ser^16^-Thr^17^-PLN (Cell Signaling, 1:1000); NCX (Millipore, 1:1000); NFATc3 (R&D Systems, 1:500) and GATA-4 (Millipore, 1:500). The proteins were measured and normalized based on the amount of protein GAPDH (Cell Signaling, 1:10000).

### Cardiovascular Measurements

#### Doppler Echocardiogram

This analysis was performed in all experimental groups (n = 10 per group) before the start of the experimental protocol and immediately after PSD (PSD96 and RT/PSD96 groups), 48 hours after the last session of resistance training for trained animals and after an equivalent number of hours for the C group.

The non-invasive cardiac function was performed by a blinded observer, using an HP Sonos 5500^®^ transducer (Philips Medical System^®^, Andover, MA, USA) with a 12 mHz transducer at a depth of 3.0 cm. The animals were anesthetized with a mixture of ketamine (50 mg/kg i.p., Dopalen, VetBrands) and xylazine (10 mg/kg, i.p., Anasedan, VetBrands). The rats were imaged in the left lateral decubitus position with three electrodes placed on their paws for the electrocardiogram. Briefly, the 2-dimensional and M-mode images from the parasternal longitudinal, transverse and apical views were obtained and recorded on a 0.5-inch videotape, and the imaging analysis and measurements were performed off-line. The following data were acquired and analyzed: E wave: maximum protodiastolic mitral flow velocity; A wave: maximum telediastolic mitral flow velocity; E/A: ratio between E and A waves; EDT: E-wave deceleration time; LA: left atrium diameter; LVAWd: diastolic LV anterior wall thickness; LVPWd: diastolic LV posterior wall thickness; LVSA: LV systolic area; LVDA: LV diastolic area and LVEF: LV ejection fraction.

#### Hemodynamic Study

The hemodynamic evaluation was performed only at the end of the protocols (n = 10 per group), on a heated operating table (37°C), under adjusted anesthesia (urethane chloride, 0.3 ml/100 g, i.p., Sigma–Aldrich^®^, St. Louis, Mo, USA) and oxygen-enriched ventilation with a closed chest. The left femoral vein was accessed for drug or saline administration. A micromanometer (MikroTip^®^ 2F; Millar Instruments Inc., Houston, Texas, USA) was inserted from the right carotid artery into the LV cavity to assess the intraventricular pressure, and a flow ultrasound probe (Transonic Systems Inc., Ithaca, NY, USA) was positioned around the ascending aorta, after a brief right thoracotomy, to estimate the LV ejection. Subsequently, bilateral vagotomy was performed to eliminate ANS interference and anesthesia bradycardic reflex. The following data were acquired and analyzed (LabChart^®^ 7 Pro—ADInstruments, Australia): the LV systolic (LVSP) and end-diastolic pressures (EDP), the rate of change of LV pressure (dP/dt), HR, cardiac index (CI), stroke volume index (SVI) and stroke work index (SWI).

### Statistical Analysis

Statistica 12 (StatSoft Inc., Tulsa, USA) was used for all statistical analyses. The data are presented as the mean ± standard deviation. The distribution of the data was assessed by the Shapiro Wilk’s test. Student’s t test was used to compare the plantaris muscle between the C and RT groups. For the other variables, one-way or two-way analysis of variance (ANOVA) complemented by Duncan’s post hoc test were performed. The level of statistical significance was set at *p* ≤ 0.05.

## Results

### Resistance Training

**Fig 1:**
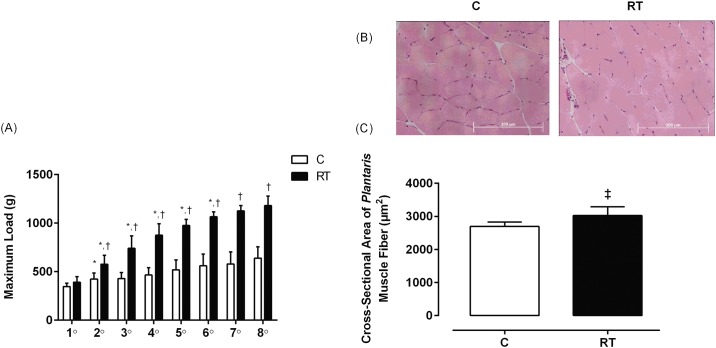
Resistance training responses in the maximum load test and the CSA of the *Plantaris* muscle fiber. (A) Evolution of maximum load (g) of the C (n = 5) and RT (n = 6) groups during 8 weeks of the RT protocol. Repeated measure ANOVA followed by Duncan’s post hoc. (B) Representative images (40x magnification) of *Plantaris* muscle fibers stained with HE from the C (n = 5) and RT (n = 6) groups. (C) CSA of *Plantaris* muscle fiber calculated from the HE-stained posterior sections using Student’s t tests for independent samples. The data are presented as the mean ± standard deviation, significance accepted: p ≤ 0.05. *—Different than the previous maximum load in the same group; ^†^—Different than the maximum load in the C group at the same time; ‡—Different from the C group.

Fig 1 show the resistance training responses. In Fig 1A, the maximum load in the RT group increased every week from the 2^nd^ to the 6^th^ week (F_(7,63)_ = 29.76, p = 0.001) in comparison with the C group. The maximum load test was increased in the C group only from the 1^st^ to the 2^nd^ week. In Fig 1B and 1C, the *Plantaris* muscle CSA was higher in the RT group compared to the C group (t = -2.51, df = 8, p = 0.03). These results indicate that the resistance training protocol was efficient in promoting skeletal muscle remodeling.

### Variation of Body Weight during 8 weeks of Resistance Training and 96 h of Sleep Deprivation

The body weight (BW) was evaluated throughout 8 weeks, as well as its Δ (final weight–initial weigth), in order to measure the gain or loss in weight during this period. As shown in Table 1, we can see the significant differences regarding time (F_(7,245)_ = 105.03, p = 0.001). Groups C, RT and PSD96 presented body weight variations throughout the weeks. Regarding Δ, RT and RT/PSD96 groups showed body weight loss when compared to C and PSD96 groups (F_(3,30)_ = 9.83, p = 0.001).

**Table 1:** Changes in body weight during 8 weeks of resistance training.

	Week 1	Week 2	Week 3	Week 4	Week 5	Week 6	Week 7	Week 8	Δ (g)
**C**	364±27	377±29*	384±29	398±29*	406±30	416±33	425±35	429±39	70±15
**RT**	382±41	394±48*	398±48	403±49	407±49	419±50*	420±45	419±40	38±11^†^^,^^‡^
**PSD96**	372±35	381±38	387±38	398±40*	409±39	418±40	425±34	425±30	62±13
**RT/PSD96**	371±23	374±22	382±22	393±22	401±27	404±26	407±25	405±26	39±20^†^^,^^‡^

Repeated Measure ANOVA with post hoc Duncan Test. The data are expressed in gram and presented as the mean ± standard deviation, significance accepted: p ≤ 0.05. N = 10.

*—Different from the previous week in the same group

^†^—Different from the C group

^‡^—Different from the PSD96 group

Δ value: weight variation (calculated by the equation: weight of week 8 –weight of week 1). This analysis was realized with one way ANOVA with post hoc Duncan Test. Note: during the training period, the C and PSD96 groups remained in their home box and received no intervention.

**Fig 2:**
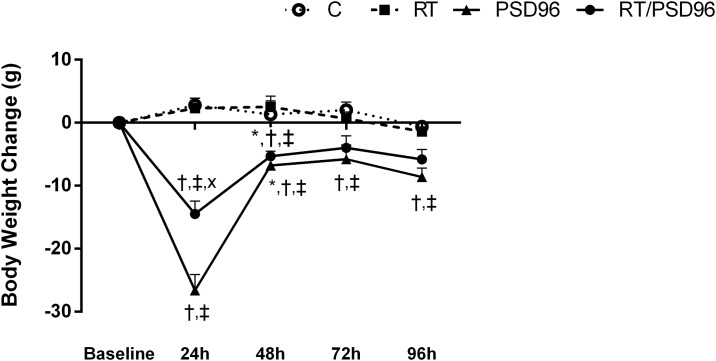
Body weight variation throughout 4 days of paradoxical sleep deprivation. Body weight change (g) of the C (n = 10), RT (n = 10), PSD96 (n = 10), RT/PSD96 (n = 10) groups during 4 days of PSD. The variation was calculated by the equation: current weight—previous weight. Repeated measure ANOVA followed by Duncan’s post hoc. The data are presented as the mean ± standard deviation, significance accepted: p ≤ 0.05. *—Different from the previous body weight in the same group; ^†^—Different from the C group at the same time; ^‡^—Different from the RT group at the same time; ^x^—Different from the PSD96 group at the same time.

Forty-eight hours after the last training session, the PSD96 and RT/PSD96 groups were submitted to SD. Every day of this protocol the body weight was evaluated for calculated the variation throughout 4 days, by the equation: current weight–previous weight (Fig 2). While the Δ of the C group did not vary significantly, the PSD96 and RT/PSD96 groups had negative Δ during 96 hours, indicating weight loss. Drastic weight loss occurred during the first 24 hours for both groups submitted to PSD, but the reduction in body mass in the RT/PSD96 group was milder. The ΔBW was reduced at 72 and 96 hours for both groups, which indicates that the animals adapted to the experimental procedure.

### Hormone Profiling

In Table 2, IGF-1 (F_(3,11)_ = 11.968, p = 0.001) and total testosterone (F_(3,35)_ = 17.030, p = 0.001) were increased in the RT group and were decreased in the PSD96 group compared to the C group. The RT/PSD96 group had similar levels of these hormones compared to the C group. The plasma corticosterone level was increased in the PSD96 and RT/PSD96 groups compared to the C group. However, the RT/PSD96 group had lower levels compared to the PSD96 group (F_(3,35)_ = 16.087, p = 0.001). T_4_ was lower in the RT/PSD96 compared to the RT group (F_(3,36)_ = 5.1489, p = 0.004). The T_3_ (p = 0.30) and CRP (p = 0.50) were similar among the groups. The Ang II measurements was increased in the PSD96 and RT/PSD96 groups compared to the C group. RT/PSD96 group was also different from RT group (F_(3,35)_ = 5.54, p = 0.004).

**Table 2:** Biochemical variables.

Variables	C	RT	PSD96	RT/PSD96
**IGF-1 (pg/mL)**	292±66	512±106*	149±34*^.^^†^	365±130^†^^.^^‡^
**Testosterone Total (ng/dL)**	231±67	423±109*	146±31*^.^^†^	275±110^†^^.^^‡^
**Corticosterone (ng/mL)**	24±17	27±25	192±113*^.^^†^	114±46*^.^^†^^.^^‡^
**T**_**3**_ **(ng/mL)**	59±14	62±14	65±14	69±9
**T**_**4**_ **(ng/dL)**	6.5±2.4	7.6±1.2	5.0±1.4^†^	5.3±1.0^†^
**CRP (mg/dL)**	0.28±0.09	0.27±0.08	0.31±0.07	0.32±0.05
**Angiotensin II (pg/mL)**	12.93±3.69	14.51±1.39	18.04±3.68*	19.38±3.58*^†^

One way ANOVA followed by Duncan’s post hoc. The data are shown as the mean ± standard deviation, significance accepted: p ≤ 0.05. N = 10.

*—Different from the C group

^†^—Different from the RT group

^‡^—Different from the PSD96 group

### Heart Morphometric Study

According to the left atrium index of the groups: C (0.62±0.09 mg/mm); RT (0.60±0.13 mg/mm); PSD96 (0.78±0.04 mg/mm) and RT/PSD96 (0.65±0.11 mg/mm); we only observed that this was higher in the PSD96 group compared to C group, indicating left atrium hypertrophy (F_(3,17)_ = 3,2120, p = 0.02).

**Fig 3:**
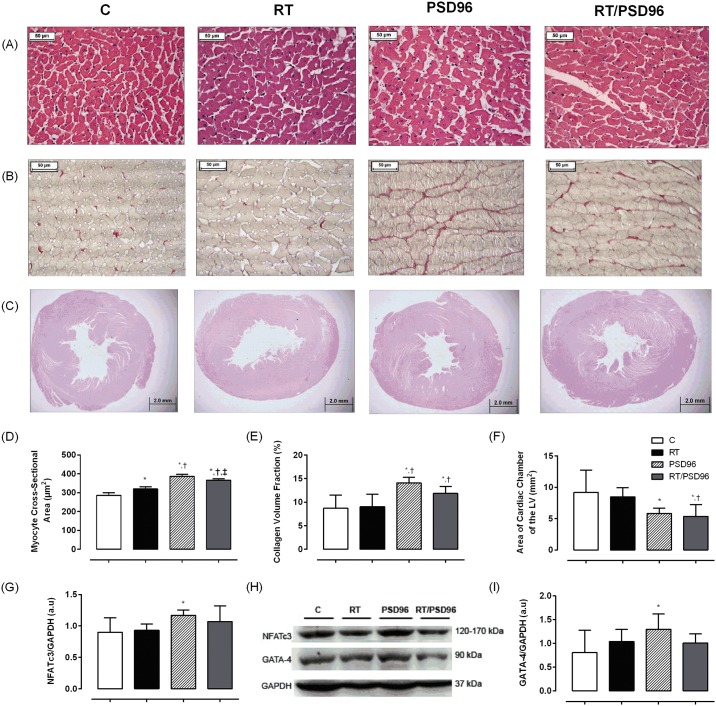
Ventricular remodeling, fibrosis and expression of proteins involved in the pathologic cardiac hypertrophy pathway. Representative images (40x magnification) of HE-stained cross sections (A) of the LV of rats without manipulation (C; n = 6) or submitted to RT (RT; n = 6) or PSD (PSD96; n = 6) or RT followed by PSD (RT/PSD96; n = 6). (B) The images (40x magnification) are the LV tissue sections stained with picrosirius red. Red color stretches are collagen depositions. (C) Images (1.25x magnification) of HE-stained the LV cavity diameter of the heart. (D) Myocyte CSA calculated from the HE-stained sections as shown in A. (E) Histogram showing collagen volume fraction in the LV tissues. (F) Results of the analysis of the LV cavity diameter as shown in C. (G) Quantification of NFATc3 expression. (H) Representative blots of NFATc3, GATA-4 and GAPDH. (I) Quantification of GATA-4 expression. For analysis, we utilized one way ANOVA followed by Duncan’s post hoc. The data are shown as the mean ± standard deviation, significance accepted: p ≤ 0.05. *—Different from the C group; ^†^—Different from the RT group; ^‡^—Different from the PSD96 group.

The LV cardiomyocytes CSA were higher in the RT, PSD96 and RT/PSD96 groups when compared to the C group (Fig 3A and 3D). However, this increase was more expressive in PSD96 group, which was differ from RT and RT/PSD96 groups. These latter were also different from each other (F_(3,19)_ = 98.841, p = 0.001).

The collagen volume fraction of the myocardium was higher in the PSD96 and RT/PSD96 groups compared to the C or RT groups (F_(3,18)_ = 8.0797, p = 0.001), (Fig 3B and 3E).

Additionally, a reduction in the LV cavity diameter was observed in the PSD96 (p = 0.02) and RT/PSD96 (p = 0.01) groups when compared with the C group (Fig 3C and 3F). The RT/PSD96 group was also different from the RT group (p = 0.03) (F_(3,17)_ = 4.0898, p = 0.02).

### Western Blotting

#### Expression of Proteins Involved in the Pathologic Cardiac Hypertrophy Pathway

In Fig 3G, 3H and 3I we can see that both proteins involved in this pathway, NFATc3 (F_(3,16)_ = 2.22, p = 0.05) and GATA-4 (F_(3,15)_ = 1.60, p = 0.05), were higher in the PSD96 group compared to C group, suggesting pathologic cardiac hypertrophy.

#### Expression of Proteins Involved the Maintenance of Normal Cardiac Ca^2+^ Homeostasis

**Fig 4:**
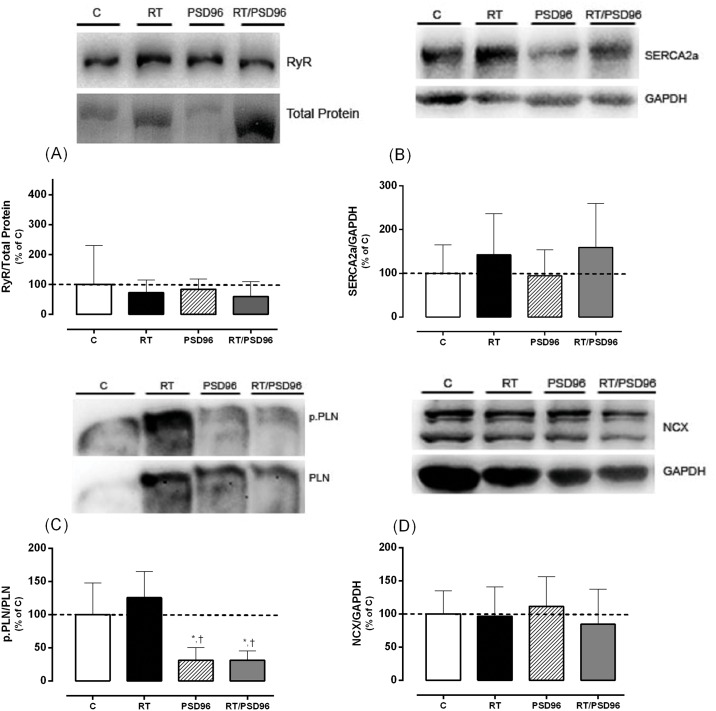
Expression of Proteins Involved the Maintenance of Normal Cardiac Ca^2+^ Homeostasis. (A) Representative blot of RyR and their normalization for total protein and the demonstrative graph of statistical analysis. (B) Representative blot of SERCA2a and their normalization for GAPDH and the demonstrative graph of statistical analysis. (C) Representative blot of phospho-Ser^16^-Thr^17^-PLN (p.PLN) and their normalization for total PLN and the demonstrative graph of statistical analysis. (D) Representative blot of NCX and their normalization for GAPDH and the demonstrative graph of statistical analysis. For analysis, we utilized one way ANOVA followed by Duncan’s post hoc. The data are shown as the mean ± standard deviation, significance accepted: p ≤ 0.05. *—Different from the C group; ^†^—Different from the RT group.

In Fig 4A, 4B and 4D, we observed that RyR (F_(3,16)_ = 0.263, p = 0.80); SERCA2a (F_(3,20)_ = 0.917, p = 0.40) and NCX (F_(3,20)_ = 0.37, p = 0.70) did not change among the groups. However, the ratio of phospho-PLN to total PLN (Fig 4C) was decreased in both groups submitted the PSD when compared to C and RT groups (F_(3,16)_ = 11.57, p = 0.001), suggesting a reduction of the active form of this protein and consequently diastolic dysfunction.

### Doppler Echocardiogram

Table 3 shows that the RT group had lower E waves after the resistance training protocol. The comparison of the E waves obtained at the end among groups found higher values in the RT/PSD96 group compared to the RT group (F_(3,20)_ = 4.488, p = 0.01). Similarly, the post protocol A wave increased in the PSD96 and RT/PSD96 groups compared to the C and RT groups, and the PSD96 group was also different from its baseline (F_(3,20)_ = 3.502, p = 0.03). EDT was higher post protocol (F_(3,26)_ = 7.454, p = 0.006) in the RT group compared to baseline. In the PSD96 group, the inverse behavior was observed and characterized by a reduction post protocol compared to baseline and the C group, which also differed from the RT/PSD96 group.

**Table 3:** Echocardiographic variables.

	C		RT		PSD96		RT/PSD96	
Variables	Before	After	Before	After	Before	After	Before	After
**E wave (cm/s)**	86±11	88±12	94±5.0	77±16*	91±13	96±14^‡^	90±6.6	96±10^‡^
**A wave (cm/s)**	47±7.6	45±8.2	47±7.8	42±7.4	44±5.9	58±16*^,^^†^^,^^‡^	48±5.9	56±4.7^†^^,^^‡^
**E/A**	1.9±0.3	2.1±0.6	2.0±0.3	1.8±0.2	2.0±0.4	1.9±0.5	1.7±0.3	1.8±0.5
**EDT (ms)**	48±12	50±9.4	37±7.1	55±6.6*	55±11	39±7.7*^,^^†^^,^^‡^	41±9.3	39±10^†^^,^^‡^
**LA (cm)**	0.5±0.08	0.6±0.09*	0.6±0.06	0.7±0.07*	0.6±0.04	0.7±0.04^‡^	0.6±0.04	0.6±0.08
**LVAWd (cm)**	0.16±0.008	0.16±0.01	0.15±0.009	0.17±0.009*	0.17±0.01	0.19±0.01*^,^^†^^,^^‡^	0.16±0.02	0.17±0.01^x^
**LVPWd (cm)**	0.17±0.01	0.17±0.01	0.15±0.008	0.16±0.007	0.16±0.01	0.18±0.01*^,^^‡^	0.15±0.01	0.17±0.01*
**LVSA (cm**^**2**^**)**	0.09±0.01	0.13±0.03*	0.12±0.02	0.11±0.01	0.11±0.01	0.10±0.02^†^	0.10±0.01	0.12±0.01
**LVDA (cm**^**2**^**)**	0.3±0.05	0.4±0.08*	0.4±0.04	0.4±0.08	0.4±0.03	0.4±0.06	0.4±0.02	0.5±0.02
**LVEF (%)**	67±0.8	62±0.9	60±0.5	66±0.3	63±0.4	66±0.7	67±0.4	66±0.6

Repeated Measure ANOVA with post hoc Duncan Test. The data are presented as the mean ± standard deviation, significance accepted: p ≤ 0.05. N = 10.

*—Different from the baseline of the same group

^†^—Different from the C group at the same time

^‡^—Different from the RT at the same time

^x^—Different from the PSD96 group at same time

E wave: maximum protodiastolic mitral flow velocity; A wave: maximum telediastolic mitral flow velocity; E/A: ratio between E and A waves; EDT: E-wave deceleration time; LA: left atrium diameter; LVAWd: diastolic left ventricle anterior wall thickness; LVPWd: diastolic left ventricle posterior wall thickness; LVSA: left ventricle systolic area; LVDA: left ventricle diastolic area; and LVEF: left ventricle ejection fraction.

Regarding cardiac structure, LVAWd was higher in the RT group post protocol compared to baseline and was higher in the PSD96 group compared to the C and RT/PSD96 groups (F_(3,26)_ = 5.07, p = 0.006). The LVPWd was higher in the PSD96 and RT/PSD96 groups post protocol compared to their respective baselines (F_(3,21)_ = 3.666, p = 0.02).

**Fig 5:**
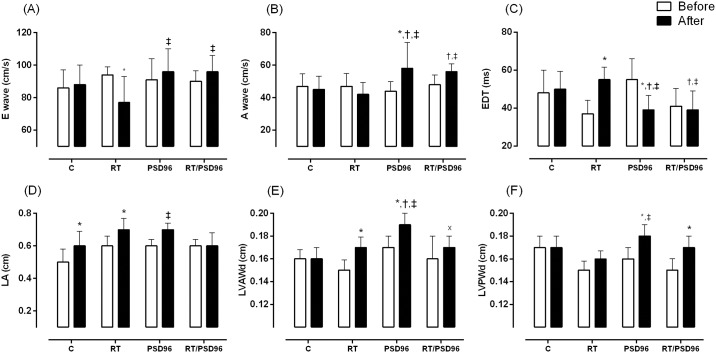
Main echocardiographic variables. Results of (A) E wave: maximum protodiastolic mitral flow velocity; (B) A wave: maximum telediastolic mitral flow velocity; (C) EDT: E-wave deceleration time; (D) LA: left atrium diameter; (E) LVAWd: diastolic left ventricle anterior wall thickness; (F) LVPWd: diastolic left ventricle posterior wall thickness. Repeated Measure ANOVA with post hoc Duncan Test. The data are presented as the mean ± standard deviation, significance accepted: p ≤ 0.05. N = 10. *—Different from the baseline of the same group; ^†^—Different from the C group at the same time; ^‡^—Different from the RT at the same time; ^x^—Different from the PSD96 group at same time.

LVSA was reduced post protocol in the PSD96 group compared to the C group (F_(3,22)_ = 1854.01, p = 0.03) and increased at the post protocol time point compared to baseline. Similarly, LVDA was higher in the C group post protocol when compared to baseline (F_(3,25)_ = 5.537, p = 0.02). The Fig 5 show the main results of this analysis.

### Hemodynamic Study

As show in Table 4, LVSP was higher in the PSD96 group compared to other groups (F_(3,30)_ = 4.1978, p = 0.01). The RT/PSD96 group had higher +dP/dt levels than the RT group (F_(3,27)_ = 5.2348, p = 0.005). The -dP/dt levels was increased in the RT/PSD96 group when compared to others (F_(3,26)_ = 2.3063, p = 0.04).

**Table 4:** Hemodynamic variables.

Variables	C	RT	PSD96	RT/PSD96
**LVSP (mmHg)**	126±9.8	122±14	147±17*^,^^†^	130±19^‡^
**LVEDP (mmHg)**	4.1±1.6	5.2±1.2	3.2±1.3	4.5±3.5
**+dP/dt (mmHg)/s**	9540±2822	7933±1625	12144±748^†^	12408±4275^†^
**-dP/dt (mmHg)/s**	-7129±908	-7163±2059	-7054±3864	-9729±892*^,^^†^^,^^‡^
**HR (beats/min)**	406±45	388±21	443±33*^,^^†^	452±18*^,^^†^
**CI (mL/kg/min)**	119±20	154±24*	161±37*	118±21^†^^,^^‡^
**SVI (mL/kg/beat)**	0.3±0.05	0.4±0.07*	0.4±0.07*	0.3±0.04^†^^,^^‡^
**SWI (g·m//beat)**	0.5±0.09	0.6±0.12	0.7±0.22*	0.5±0.15^‡^

One way ANOVA followed by Duncan’s post hoc. The data are presented as the mean ± standard deviation, significance accepted: p ≤ 0.05. N = 10.

*—Different from the C group

^†^—Different from the RT group

^‡^—Different from the PSD96 group

LVSP: left ventricular systolic pressure; LVEDP: left ventricular end-diastolic pressure; +dP/dt: maximum positive time derivative of developed pressure; -dP/dt: maximum negative derivative of developed pressure; HR: heart rate; CI: cardiac index; SVI: stroke volume index and SWI: stroke work index.

**Fig 6:**
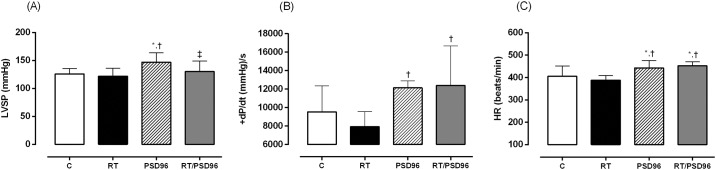
Main hemodynamic variables. Results of (A) LVSP: left ventricular systolic pressure; (B) +dP/dt: maximum positive time derivative of developed pressure; (C) HR: heart rate. One way ANOVA followed by Duncan’s post hoc. The data are presented as the mean ± standard deviation, significance accepted: p ≤ 0.05. N = 10. *—Different from the C group; ^†^—Different from the RT group; ^‡^—Different from the PSD96 group.

The HR was enhanced in the PSD96 and RT/PSD96 groups compared to the C and RT groups (F_(3,26)_ = 6.7190, p = 0.001). CI (F_(3,25)_ = 5.3321, p = 0.005) and SVI (F_(3,25)_ = 8.8011, p = 0.001) increased in the RT and PSD96 groups compared to the C and RT/PSD96 groups. SWI was higher in the PSD96 group compared to the C and RT/PSD96 groups (F_(3,27)_ = 4.2166, p = 0.01). The Fig 6 show the main results of this analysis.

## Discussion

The main results demonstrate the efficiency of the resistance training protocol, as well as the harm caused by PSD96, in the cardiovascular and hormonal scope. When resistance training was performed before PSD96, it could prevent some changes caused by PSD such as increased LVSP and hormonal dysregulation.

### Hormonal Profiling

PSD causes stimulation of sympathetic activity of the HPA axis and consequently an increase in corticosterone secretion was observed, as seen in the PSD96 group. The levels became elevated after only 24 hours of PSD [15], and remained for up to 96 hours [14,15]. This increase in plasma concentrations of glucocorticoids is directly responsible for the apoptosis of Leydig cells, and consequently for the suppression of testosterone synthesis [31,32], which was reduced in the PSD96 group in our study.

Another anabolic hormone that was reduced in our study is IGF-1, which declined by 51% in the PSD96 group when compared to the C group. Everson and Crowley [33] justify this behavior by emphasizing that animals deprived of sleep have significantly decreased GH. In contrast to PSD96 group, we found that the RT/PSD96 group did not modified its levels of IGF-1 and testosterone when compared to the C group. This profile characterizes the preventive effect of resistance training, in which testosterone and IGF-1 pathways, in a synergistic action, culminate in protein synthesis and consequently in muscle hypertrophy [25,26], which may explain the attenuation of the catabolic response observed in the RT/PSD96 group.

Regarding thyroid hormones, both groups submitted to SD protocol showed reduction in the T_4_ levels compared to the RT group, while T_3_ levels did not change. Falling levels of T_4_ was also noted by previous studies [34,35], that attributed these results to changes in the central mechanisms, which cause a lack of thyrotropin-releasing hormone (TRH) secretion and consequently affect the pituitary release of thyroid-stimulating hormone (TSH) and the thyroidal response of T_4_. Another justification for this finding is the accelerated activity of type 2 iodothyronine deiodinase enzyme in brown adipose tissue, which increased the systemic fractional conversion of T_4_ to T_3_ and consequently reduced serum T_4_ levels [36].

Another point to highlight was the behavior of CRP. Our results are compatible with most clinical studies, which also have not found increased CRP in SD conditions [37,38], while the only study with animal model showed the reduces of CRP in rats with lesions in the ventrolateral preoptic area, which spontaneously made them sleep about 30% less. This setback results should be explained by differences in protocols used, included method, duration of SD, circadian rhythm, light exposure, among others [39]. Further studies are needed to clearly understand this relationship.

Finally, referring to Ang II, we observed an increase in PSD96 and RT/PSD96 groups. This result can be explained by altering neuroendocrine function and activating renin-angiotensin system [40,41]. The Ang II potently enhances catecholamine releases [42], which are also increased in SD conditions [15]. Thus, together, these will play a crucial role in modulating cardiovascular pathology at the molecular level and consequently in their morphology and function [17,43].

### Paradoxical Sleep Deprivation

The loss of body weight was more pronounced during the first 24 hours of PSD, however, it remained different from C and RT groups up to 96 hours. These findings corroborate the results of Galvão and colleagues [44], which showed a body weight loss in the first 24 hours of PSD, distinguishing from the 48, 72 and 96 hours as well as the control group at all periods. Although body mass loss was evident in both groups submitted to PSD, this was attenuated in the first 24 hours in the group that was submitted to previous resistance training. The occurrence of this behavior can be explained by the hormonal profile minimized by resistance training, which was able to attenuate both IGF-1 and testosterone at basal levels, and decreased the level of corticosterone when compared to the PSD96 group [25].

The reduction of body weight of rats associated with SD is well-established in the literature, and it is strongly influenced by the hormonal profile derived from the condition imposed, thereby resulting in the reduction of adipose tissue [45] and also the reduction of muscle mass, leading to muscle atrophy [14,46].

Both rats and humans present a high metabolic activity followed by an increase of corticosterone [14,15] and cortisol [47] levels, respectively. In rats, this profile triggers an increase in total daily energy expenditure, i.e., negative energy balance that translates into an important weight loss, despite hyperphagia observed [45]. On the other hand, in humans, this adverse condition appears to be more closely linked to the extended wakefulness, where are observed changes in appetite hormones, with a decrease in the levels of leptin and an increase in levels of ghrelin [48,49]. Corroborating, this increase of time awake caused an increased need of energy to sustain, in addition it represents more opportunities for eating, mainly calorie-dense nutrients with high carbohydrate content, which may contribute to weight gain [48].

Regarding the method adopted, it is worth highlighting that it was given a standard period of adaptation for the animals allowing them to familiarize themselves with the platforms, and letting them know that the platforms are safe places for them to keep themselves dry. Many questions arose concerning the thermoregulatory changes in PSD protocols for rats, however information about these changes were only found for the disc-over-water method. In this method, paradoxical sleep deprived rats presented a decrease of intraperitoneal temperature, which is compensated by the increase in self-selected ambient temperature. This is a compensatory mechanism for the stress caused by PSD [50].

### Cardiac Remodeling and Molecular Function

There was hypertrophied heart tissue in all experimental groups compared with the C group. The cardiac hypertrophy observed in the RT group was possibly mediated by an increase in BP during the course of physical exercise [51–54]. This type of hypertrophy triggered by resistance training is known as physiological concentric LV hypertrophy, characterized by increased wall thickness of the heart without a reduction in cavity size [20].

In contrast, pathological concentric hypertrophy is characterized by an increase in the wall thickness of the heart with concomitant reduction in the cavity size and fibrosis presence. This response can be triggered by supraphysiological levels of glucocorticoids [17,20,43] and Ang II [20,21], by activation of Ang II type 1 (AT1) receptor, causing an increased of BP, which when sustained long-term culminates in increased production of collagen [43].

Among the molecular determinants of this response, calcineurin-NFAT pathway can be highlighted [22]. Calcineurin is a serine/threonine-specific phosphatase that is activated by the increase of intracellular Ca^2+^. This activation evokes a dephosphorylation of NFATc3 within the cytoplasm, which translocates to the nucleus, where it associates with transcription factor like GATA-4, to regulate the cardiac genes and develop cardiac hypertrophy [20,22,55].

Thus, the hypertrophy associated with heart cavity reduction and fibrosis, as observed in the PSD96 group, may indicate a pathological condition, due to increase the corticosterone, Ang II and expression of proteins like NFATc3 and GATA-4. It is worth to emphasize that our study is the first to examine the effecst of SD on cardiac morphology scope, so the relationships established to justify this important finding refers to the hormonal and proteic expression profile. Considering that many researches within this subject establish long-term protocols, unlike the protocol chosen for this study (approximated 96 hours or less), it is not possible to compare them.

In addition, regarding the molecular functioning, we observed that the phospho-Ser^16^-Thr^17^-PLN:PLN ratio decreased in PSD96 and RT/PSD96 groups. In conditions that demand arises, such as SD, the release of adrenaline is observed allowing that heart respond to stress in a few seconds [56]. Adrenaline and other hormones related to stress initiate an important signal-transduction pathway by activating the receptor in the cardiac cell membrane [17,56]. This signal can affect the function of cardiac protein related to overall cardiac function like phospho-Ser^16^-Thr^17^-PLN. The latter regulates the activity of SERCA2a whose function is Ca^2+^ uptake by the sarcoplasmic reticulum. When the PLN is in its dephosphorylated state it reduces the affinity of SERCA2a for Ca^2+^inhibiting this Ca^2+^pump activity [56]. Despite we not observed statistical significance at SERCA2a expression, the reduce at phospho-Ser^16^-Thr^17^-PLN:PLN ratio, probably triggered by higher levels of corticosterone, Ang II and adrenaline, can indicate a long-term impair of the diastolic function. Any defect in the removal Ca^2+^ would impair cardiac relaxation and consequently affect the preparation for the next contraction, i.e, systolic function [57].

### Doppler Echocardiogram

We noticed a rise in the E wave and A wave and a reduction in the EDT in the PSD96 and RT/PSD96 groups. These results are similar to those observed in animals with supravalvular aortic stenosis [58]. The increase in the A wave probably culminates in a higher velocity of transmitral flow during the end diastole. Furthermore, the evaluation of the LA is also a way to determine the severity of diastolic dysfunction [59], which in turn corroborated our morphological findings, which showed an increase in the weight of the LA in the PSD96 group. During ventricular diastole, the LA is exposed to intraventricular pressure, thus, rises in ventricular filling pressures cause an increase in atrial pressure and consequently atrial remodeling is observed [60].

Considerable changes in the diastolic thickness of the posterior and anterior walls of the LV in the PSD96 group were observed. These findings, when combined with histological data of the cavity size, resemble data from nephrectomized animals, suggesting concentric pathological cardiac hypertrophy [20].

### Hemodynamic Measurements

Under the same anesthetic plane and the bilateral vagotomy condition, we observed the reduction in LVSP in the RT group compared to other groups can be attributed to the pressure reduction effect commonly observed after resistance training. This behavior is associated with exercise intensity so, as the resistance training protocol adopted in our study was high intensity, a reduction in LVSP was observed, probably caused by a decrease in BP [23,61].

In the PSD96 group, the increase in LVSP compared with all groups demonstrates the influence of PSD on the cardiac tissue, even in the absence of stimuli from the ANS. In this context, Joukar and colleagues [11] reported that rats deprived of sleep for 72 hours showed an increase in average BP at 24 and 96 hours of PSD, which was not observed by Perry and colleagues [62]. Studies involving humans also highlight the rise of BP after SD. Sauvet and colleagues [63] observed this behavior in individuals deprived of sleep continuously for 40 hours, while Kato and colleagues [64] only observed that profile at 24 hours of SD. In this context, it was observed that sleep time below the average of between 7–8 hours is associated with an increased prevalence of hypertension [65]. On the other hand, the REM SD caused by antidepressant drugs did not show any cardiac alterations [66].

Among the explanations for this association stands out the arterial baroreflex, characterized by a loss of sensitivity to BP variation and consequently elevated BP [12,13]. This change was similar to that reported by Qian and colleagues [67] in a study of stress in an animal model and was reversed by the intracerebroventricular administration of an Ang II antagonist, suggesting that stress and consequently the baroreflex dysfunction are caused by the activation of Ang II in the central nervous system. Thus, it is possible that this mechanism is one of the factors responsible for the baroreflex resetting after SD corroborating our results.

The AT1 receptor is activated by corticosterone, which at high levels, as observed in our study, triggers a cascade of signaling in cardiomyocytes and cardiac fibroblasts culminating in fibrosis, increased oxidative stress, and consequently in functional damage of the cardiovascular system [17,43].

Another possible mechanism is the decreased expression of the GABA_A_ receptor in the paraventricular nucleus [68]. The rostroventrolateral medulla and the paraventricular nucleus of the hypothalamus are primarily involved in the control of the cardiovascular system, and so any imbalance in the excitation and inhibition of these neurons may interfere with the organization and function of neural circuits in terms of hypertension [69].

As for HR, an increase was observed in the PSD96 and RT/PSD96 groups. This profile is consistent with the view that the adjustment of BP also influences other variables such as cardiac contractility, peripheral vascular resistance and HR [70]. The parameters related to ventricular ejection, SVI and SWI, also had higher values in the PSD96 group.

As for the derivatives of ventricular pressure, these were lower in animals with cardiac dysfunction [71,72] and those that were treated long-term with synthetic glucocorticoids [43]. Our results demonstrate that both +dP/dt and -dP/dt increased significantly in the PSD96 and RT/PSD96 groups. However, this increase is not related to an improvement in the functionality of the LV. This increase is associated with the rise in the values of LVSP and HR, which together lead us to suggest a sympathetic hyperactivity associated with the neuroendocrine dysregulation generated by PSD.

Therefore, this work brings together a set of results that allows us to conclude about the importance of sleep, showing that PSD is able to trigger significant damages to the function and cardiac structure, which may be related autonomic dysfunction associated with the catabolic frame. Thus, establishing strategies such as, sleep well, exercise and eat well can contribute for attenuating or reversing the effects observed.

### Study Limitations

The present study assessed the impact of 96 hours of continuous SD on cardiac function to understand the mechanisms involved in this relationship, including resistance exercise as a strategie to improve the alterations observed. The lack of cardiovascular measurements like telemetry in freely-behaving animals, during resistance exercise and PSD protocols should also be acknowledged as a limitation. In addition, it is worth highlighiting that the study was designed to determine the effect of SD on cardiac profile (molecular, morphologic and functional) in rats in order to understand the impact of this condition in the absence of co-morbidies, which is observed in clinical studies and may confound the results. Thus, it is necessary to use caution in applying these findings in clinical investigations.

## Conclusion

In conclusion, PSD96 had a negative impact on cardiac morphofunction and on the hormonal axis, and resistance training effectively attenuated the main alterations observed after PSD.

## Supporting Information

S1 Dataset General Data. (XLS)

S2 Dataset Doppler Echocardiography. (XLS)

S3 Dataset Hemodynamic. (XLS)

S1 Fig Device used in the resistance training protocol. Rat with a weight cylinder attached to its tail climbing the ladder. On the top of the ladder, there is a shelter for the rats to rest between sets.
(JPG)
