# Supplementary figures and images for: Paradoxical Sleep Deprivation Causes Cardiac Dysfunction and the Impairment Is Attenuated by Resistance Training
Source: PLoS One. 2016 Nov 23;11(11):e0167029. doi: 10.1371/journal.pone.0167029 (PMC5120843; doi:10.1371/journal.pone.0167029)

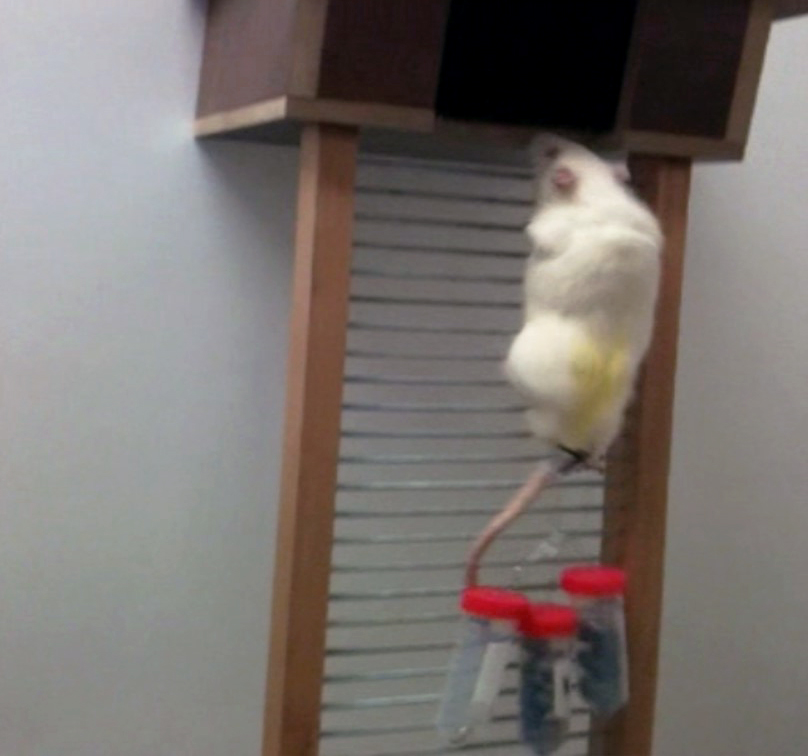

Supplement: S1 Fig — Rat with a weight cylinder attached to its tail climbing the ladder. On the top of the ladder, there is a shelter for the rats to rest between sets. (JPG) [file pone.0167029.s004.jpg]
